# Impact of Concomitant Tonsillectomy on the Management and Outcomes of Pediatric Adenoidectomy

**DOI:** 10.1007/s12070-024-05217-2

**Published:** 2024-11-22

**Authors:** Aman M. Patel, Lucy Revercomb, Ariana L. Shaari, Vraj P. Shah

**Affiliations:** Department of Otolaryngology - Head and Neck Surgery, Rutgers New Jersey Medical School, 185 S Orange Ave Newark, Newark, NJ 07103 USA

**Keywords:** Adenoidectomy, Tonsillectomy, Pediatric, Management, Outcomes

## Abstract

Adenoidectomy is a common otolaryngologic procedure that may be performed with concomitant tonsillectomy (T&A). This study investigates differences between pediatric inpatients undergoing adenoidectomy alone and T&A. The 2016 Kid’s Inpatient Database was used to identify pediatric patients undergoing adenoidectomy (ICD-10: 0CTQXZZ) with and without tonsillectomy (ICD-10: 0CTP, 0CBP). Common comorbidities, postoperative complications, and procedures undergone were identified via ICD-10 codes. Univariate and multivariable analyses were performed to determine statistical associations with tonsillectomy status. Of the 5,540 inpatients who met inclusion criteria, the majority underwent T&A (88.9%). Mean patient age was 4.9 years. On multivariable analysis adjusting for patient demographics, hospital data, and severity of illness, T&A patients had similar total charges ($35,442 vs. $48,577, *p* = 0.880), length of stay (LOS) (2.5 vs. 3.9 days, *p* = 0.070), and number of procedures undergone (3.3 vs. 3.8 procedures, *p* = 0.884) as adenoidectomy alone patients. T&A patients had lower odds for undergoing ear, nose, sinus, mouth, or throat inspection (OR 0.59, 95% CI 0.46–0.75) and drainage (OR 0.56, 95% CI 0.46–0.68), bronchoscopy (OR 0.57, 95% CI 0.44–0.75), laryngoscopy (OR 0.64, 95% CI 0.49–0.83), and imaging (OR 0.56, 95% CI 0.32–0.996) than adenoidectomy alone patients (*p* < 0.05). T&A patients had higher odds for undergoing control of bleeding in the head and neck (OR 8.09, 95% CI 1.49–43.91, *p* < 0.001). Odds for undergoing ear, nose, sinus, mouth, or throat inspection and drainage, bronchoscopy, laryngoscopy, imaging, and control of bleeding in the head and neck varied by tonsillectomy status.

## Introduction

The adenoid is a mass of nasopharyngeal lymphoid tissue that frequently serves as a reservoir for respiratory pathogens and may obstruct the nasal airway [1–5]. Otolaryngologists have commonly performed adenoidectomies in pediatric patients to manage adenoid hypertrophy, obstructive sleep apnea, sleep disordered breathing, and otitis media with effusion [4, 6]. Patients with recurrent otitis media, rhinosinusitis, or purulent rhinorrhea may also benefit from adenoidectomy [1, 6–8].While evidence supporting the long-term efficacy of adenoidectomy remains limited, removing the adenoid in very young children may negatively influence the early development of immunity [7]. Other complications of adenoidectomy include bleeding, velopharyngeal insufficiency, tubaric stenosis, and nasopharyngeal stenosis [6].

Adenoidectomy is usually performed with concomitant tonsillectomy (T&A) [9, 10]. The indications for T&A are similar to those for adenoidectomy and include sleep disordered breathing, obstructive sleep apnea, and recurrent tonsillitis [9, 11–13]. T&A has also been associated with several postoperative complaints and complications including sore throat, dysphagia, fever, dehydration, bleeding, uvular edema, and in rare cases, atlanto-axial subluxation (Grisel’s syndrome), cervical necrotizing fasciitis, cervical emphysema, and cervical osteomyelitis [14, 15]. Whether patients undergoing adenoidectomy alone have higher incidence of these complications remains unclear.

To the best of our knowledge, there exists little evidence comparing the management and outcomes of patients undergoing either adenoidectomy alone or T&A. Further investigation of the hospital course of these two common otolaryngological interventions thus has clinical utility. The objective of this study is to describe the impact of concomitant tonsillectomy on the management of adenoidectomy in pediatric patients through a retrospective analysis of a large national inpatient pediatric database: the Kids’ Inpatient Database (KID).

## Materials and Methods

### Data Source

The 2016 KID was used retrospectively to investigate the impact of concomitant tonsillectomy on adenoidectomy in pediatric inpatients. KID is sponsored by the Agency for Healthcare Research and Quality and is part of the Healthcare Cost and Utilization Project (HCUP) [16]. This database provides encounter-level and hospital-level healthcare data from patients admitted in 2016 [16]. Currently, KID is the largest publicly available database containing inpatient pediatric cases (less than 21 years old) within the United States (US) [16]. Patient diagnoses and procedure information are included in the database using *ICD-10* (*International Classification of Diseases*,* Tenth Revision*) codes. This study was granted exemption from institutional review board approval at Rutgers New Jersey Medical School because patient cases in KID are fully deidentified.

### Cohort and Variable Selection

To select our cohort, we queried KID for inpatients with an *ICD-10* diagnosis code corresponding to adenoidectomy (0CTQXZZ). KID consists of patient demographic data, such as age, sex (male and female), race (White, Black, Hispanic, Asian and Pacific Islander, Native American, and other), income quartile relative to the median income in the patient’s ZIP code, primary payer status (Medicare, Medicaid, private insurance, self-pay, no charge, and other), hospital region within the US (Northeast, South, Midwest, and West), and hospital location and teaching status (rural non-teaching, urban non-teaching, and urban teaching). KID also identifies patient severity of illness at time of admission as defined by loss of function (All Patient Refined Diagnosis Related Groups). Although KID does not directly report comorbidities, *ICD-10* codes were used to extrapolate comorbidities within the cohort. The most common comorbidities (*ICD-10*) were sleep apnea (G473), otitis media (H65-H67), asthma (J45), overweightness and obesity (E66), gastroesophageal reflux disease (GERD) (K21), and Down syndrome (Q90).

Total charges, length of stay, number of procedures undergone during inpatient stay, time from admission until first procedure, and mortality are also reported in KID and were included in our analysis. The most common classes of procedures (ICD-10) performed in our cohort were included in analysis: drainage procedure of the ear, nose, sinus, mouth, or throat (099, 0C9), inspection procedure of the ear, nose, sinus, mouth, or throat (09 J, 0CJ), repair procedure of the ear, nose, sinus, mouth, or throat (09Q, 0CQ), bronchoscopy (0BJ08ZZ), laryngoscopy (0CJS8ZZ), and imaging (B). Several complications were included in analysis: control of bleeding in the head and neck (0W30-0W36), ventilation and intubation (5A09, 0BH17EZ), and orbital and intracranial complications (A39.0, H05, G00-G01, G03.9, G04.2, G06, G08-G09).

### Statistical Analyses

Per the protocol outlined in HCUP, discharge weights were applied to the cohort to provide representative national estimates. All results are reported as nationally weighted estimates. All variables were compared by tonsillectomy status via univariate analysis. Pearson chi-square test and independent samples t-test were each used when appropriate. A multivariable analysis was then performed for each disease management and outcome variable to identify the independent impact of tonsillectomy status while accounting for the following covariables: age, sex, race, income status, primary payer status, hospital region, hospital location and teaching status, and severity of illness. Multivariable analysis results are reported with adenoidectomy alone patients as the reference. Multivariable logistic and linear regressions were used when appropriate.

All data obtained from KID were exported and analyzed with SPSS version 25.0 (IBM Corp, Armonk NY). Statistical significance was assessed at a 2-sided alpha value of 0.05. The standard error of the mean, odds ratio (OR), and 95% confidence interval (CI) are reported where appropriate. Marginal values are reported as the adjusted difference in values between T&A and reference adenoidectomy alone cohorts for continuous variables in multivariable linear regression. ORs are reported for categorical variables in multivariable logistic regression.

## Results

A total of 5,540 inpatients undergoing adenoidectomy were included in this analysis. Concomitant tonsillectomy was identified in 4,927 (88.9%) patients. The majority of patients were male (60.7%), had Medicaid coverage (60.6%), and were treated at urban teaching hospitals (92.2%). Mean age for all patients was 4.9 years. Mean ages of T&A and adenoidectomy alone patients were 5.1 and 3.5 years, respectively. Age, race, primary payer status, hospital region, hospital location and teaching status, and severity of illness varied significantly by concomitant tonsillectomy (*p* < 0.005) (Table 1). T&A patients had higher incidence of sleep apnea, obesity, and tonsillar hypertrophy but lower incidence of otitis media, GERD, breathing abnormalities, developmental delay, adenoid hypertrophy, aphagia or dysphagia, congenital laryngomalacia, nasal congestion, atrial septal defect, ventricular septal defect, deviated nasal septum, cough, congenital tracheomalacia, and cystic fibrosis than adenoidectomy alone patients (*p* < 0.001) (Table 2). T&A patients and adenoidectomy alone patients had similar incidence of Down syndrome (*p* = 0.965). The majority of T&A patients had a primary procedure of tonsillectomy (51.9%) while the majority of adenoidectomy alone patients had a primary procedure of adenoidectomy (65.0%).

**Table 1:** Demographic data of pediatric adenoidectomies by tonsillectomy status

		Adenoidectomy Alone	Tonsillectomy & Adenoidectomy	Total	*p*-value
		*n* = 613 (11.1%)	*n* = 4927 (88.9%)	*n* = 5540	
Age	Age, years (mean [SE])	3.50 [0.16]	5.12 [0.06]	4.94 [0.06]	**< 0.001**
Sex	Male	59.6%	60.8%	60.7%	0.571
	Female	40.4%	39.2%	39.3%	
Race	White	44.8%	37.4%	38.2%	**< 0.001**
	Black	28.2%	25.3%	25.6%	
	Hispanic	17.0%	26.6%	25.6%	
	Asian- Pacific Islander	2.9%	3.7%	3.6%	
	Native American	0.5%	0.6%	0.6%	
	Other	6.5%	6.5%	6.5%	
Median Income Quartile – Patient ZIP Code	0–25%	33.9%	33.4%	33.4%	0.702
	26–50%	23.6%	24.7%	24.6%	
	51–75%	21.1%	22.2%	22.1%	
	76–100%	21.4%	19.7%	19.9%	
Primary Payer Status	Medicare	0.0%	0.3%	0.2%	**0.004**
	Medicaid	57.0%	61.0%	60.6%	
	Private Insurance	36.8%	33.8%	34.2%	
	Self-Pay	1.0%	1.4%	1.4%	
	No Charge	0.2%	0.0%	0.0%	
	Other	5.1%	3.5%	3.6%	
Hospital Region	Northeast	24.5%	23.5%	23.6%	**< 0.001**
	Midwest	21.7%	21.8%	21.8%	
	South	37.0%	28.8%	29.7%	
	West	16.8%	25.9%	24.9%	
Hospital Location & Teaching Status	Rural	1.1%	1.7%	1.6%	**0.002**
	Urban, Nonteaching	3.1%	6.6%	6.2%	
	Urban, Teaching	95.8%	91.8%	92.2%	
Severity of Illness (Loss of Function)	Minor LOF	31.5%	54.5%	51.9%	**< 0.001**
	Moderate LOF	38.2%	30.8%	31.6%	
	Major LOF	27.1%	11.7%	13.4%	
	Extreme LOF	3.1%	3.1%	3.1%	

*Note* Bolded values indicate statistical significance (*p* < 0.05)

**Table 2:** Clinical factors in pediatric adenoidectomies by tonsillectomy status

	Adenoidectomy Alone	Tonsillectomy & Adenoidectomy	Total	*p*-value
Sleep Apnea	58.5%	81.2%	78.7%	**< 0.001**
Obstructive Sleep Apnea	53.3%	75.8%	73.3%	**< 0.001**
Otitis Media	33.1%	21.7%	22.9%	**< 0.001**
Asthma	20.4%	19.0%	19.2%	0.422
Mental Health Diagnosis	19.1%	15.9%	16.3%	**0.046**
Overweight or Obese	3.4%	12.6%	11.6%	**< 0.001**
Gastro-esophageal Reflux Disease	22.8%	9.2%	10.7%	**< 0.001**
Breathing Abnormalities	16.2%	9.6%	10.3%	**< 0.001**
Developmental Delay	17.5%	9.2%	10.1%	**< 0.001**
Adenoid Hypertrophy	58.9%	2.7%	8.9%	**< 0.001**
Down Syndrome	6.9%	6.9%	6.9%	0.965
Hypoxemia	7.3%	6.6%	6.7%	0.499
Anemia	9.5%	6.2%	6.6%	**0.002**
Aphagia or Dysphagia	12.3%	5.5%	6.3%	**< 0.001**
Respiratory Complications	7.0%	6.0%	6.1%	0.321
Fluid and Electrolyte Disorders	5.4%	5.4%	5.4%	0.978
Tonsil Hypertrophy	0.8%	5.9%	5.4%	**< 0.001**
Nasal Turbinate Hypertrophy	6.2%	4.5%	4.7%	0.055
Respiratory Failure	5.4%	4.2%	4.3%	0.175
Long-Term Drug Therapy (current)	7.7%	3.7%	4.1%	**< 0.001**
Acute Tonsillitis	1.3%	4.3%	4.0%	**< 0.001**
Chronic Tonsillitis	0.2%	3.3%	2.9%	**< 0.001**
Nasal Congestion	4.1%	1.5%	1.8%	**< 0.001**
Acute Pharyngitis	0.8%	1.4%	1.3%	0.263
Chronic Adenoiditis	6.7%	0.4%	1.1%	**< 0.001**
Deviated Nasal Septum	2.1%	0.5%	0.7%	**< 0.001**
Cough	2.0%	0.6%	0.7%	**< 0.001**
Cystic Fibrosis	2.3%	0.3%	0.5%	**< 0.001**
First Procedure of Tonsillectomy	0.0%	51.9%	46.2%	**< 0.001**
First Procedure of Adenoidectomy	65.0%	38.0%	40.9%	**< 0.001**
Respiratory Congenital Malformations	18.3%	4.5%	6.0%	**< 0.001**
Congenital Laryngomalacia	15.7%	3.4%	4.8%	**< 0.001**
Congenital Tracheomalacia	2.4%	0.5%	0.7%	**< 0.001**
Circulatory Congenital Malformations	9.5%	4.0%	4.6%	**< 0.001**
Atrial Septal Defect	4.6%	2.0%	2.3%	**< 0.001**
Ventricular Septal Defect	2.3%	0.8%	0.9%	**< 0.001**

*Note* Bolded values indicate statistical significance (*p* < 0.05)

On univariate analysis, T&A patients had lower total charges ($35,442 vs. $48,577, *p* = 0.007), number of procedures undergone (3.3 vs. 3.8 procedures, *p* < 0.001), shorter time from admission to first procedure (0.3 vs. 0.9 days, *p* = 0.036), and length of stay (LOS) (2.5 vs. 3.9 days, *p* = 0.013) than adenoidectomy alone patients (Table 3). During their hospital stays, T&A patients had lower incidence of undergoing ear, nose, sinus, mouth, or throat drainage (23.2% vs. 37.4%), inspection (10.8% vs. 22.2%), and repair (1.8% vs. 4.9%) than adenoidectomy alone patients (*p* < 0.001). T&A patients also had lower incidence of undergoing bronchoscopy (7.7% vs. 17.3%), laryngoscopy (8.9% vs. 18.3%), and imaging (1.3% vs. 3.4%) and having orbital or intracranial complications (0.1% vs. 1.3%) than adenoidectomy alone patients (*p* < 0.001). T&A patients had higher incidence of undergoing control of bleeding in the head and neck (1.9% vs. 0.2%, *p* = 0.001) but similar mortality (0.04% vs. 0.0%, *p* = 0.618) and incidence of undergoing ventilation and intubation (5.1% vs. 5.2%, *p* = 0.911) and transfusion (1.4% vs. 1.5%, *p* = 0.893) as adenoidectomy alone patients.

**Table 3:** Management, charges, and outcomes of pediatric adenoidectomies by tonsillectomy status

		Adenoidectomy Alone	Tonsillectomy & Adenoidectomy	Total	*p*-value
Total Charges	Charges ($) (mean [SE])	48,576.81 [4,511.93]	35,441.51 [1,789.46]	36,901.93 [1,668.56]	**0.007**
Length of Stay	Number of Days (mean [SE])	3.94 [0.57]	2.50 [0.09]	2.66 [0.10]	**0.013**
Number of Procedures	Number of Procedures (mean [SE])	3.77 [0.11]	3.33 [0.03]	3.38 [0.03]	**< 0.001**
Time Until 1st Procedure	Number of Days (mean [SE])	0.92 [0.28]	0.32 [0.06]	0.39 [0.06]	**0.036**
Mortality	Mortality (%)	0.0%	0.04%	0.04%	0.618
Drainage: Ear, Nose, Sinus, Mouth, Throat	Procedure (%)	37.4%	23.2%	24.8%	**< 0.001**
Inspection: Ear, Nose, Sinus, Mouth, Throat	Procedure (%)	22.2%	10.8%	12.1%	**< 0.001**
Laryngoscopy	Procedure (%)	18.3%	8.9%	10.0%	**< 0.001**
Bronchoscopy	Procedure (%)	17.3%	7.7%	8.8%	**< 0.001**
Ventilation and Intubation	Procedure (%)	5.2%	5.1%	5.1%	0.911
Repair: Ear, Nose, Sinus, Mouth, Throat	Procedure (%)	4.9%	1.8%	2.1%	**< 0.001**
Control of Bleeding in Head and Neck	Procedure (%)	0.2%	1.9%	1.8%	**0.001**
Removal: Ear, Nose, Sinus, Mouth, Throat	Procedure (%)	2.9%	1.5%	1.6%	**0.006**
Imaging	Procedure (%)	3.4%	1.3%	1.5%	**< 0.001**
Transfusion	Procedure (%)	1.5%	1.4%	1.4%	0.893
Postprocedural Respiratory Hemorrhage	Complication (%)	0.5%	1.4%	1.3%	0.053
Orbital or Intracranial Complications	Complication (%)	1.3%	0.1%	0.2%	**< 0.001**

*Note* Bolded values indicate statistical significance (*p* < 0.05)

On multivariable analyses, adjusting for age, sex, race, household income, primary payer status, hospital region, hospital location and teaching status, and severity of illness, total charges (adjusted *p* = 0.880), LOS (adjusted *p* = 0.070), and number of procedures undergone (adjusted *p* = 0.884) were not significant with tonsillectomy status (Table 4). Time until first procedure remained significant (adjusted *p* = 0.044). T&A patients had lower odds for undergoing ear, nose, sinus, mouth, or throat inspection (OR 0.59, 95% CI 0.46–0.75), drainage (OR 0.56, 95% CI 0.46–0.68), and repair (OR 0.44, 95% CI 0.28–0.72) than adenoidectomy alone patients (*p* < 0.001). T&A patients also had lower odds for undergoing bronchoscopy (OR 0.57, 95% CI 0.44–0.75, *p* < 0.001), laryngoscopy (OR 0.64, 95% CI 0.49–0.83, *p* < 0.001), and imaging (OR 0.56, 95% CI 0.32–0.996, *p* = 0.049) but higher odds for undergoing ventilation and intubation (OR 1.61, 95% CI 1.03–2.50, *p* = 0.037) and control of bleeding in the head and neck (OR 8.09, 95% CI 1.49–43.91, *p* = 0.015) than adenoidectomy alone patients. T&A patients had lower odds for having orbital or intracranial complications (OR 0.06, 95% CI 0.01–0.26, *p* < 0.001) than adenoidectomy alone patients. T&A patients and adenoidectomy alone patients had similar odds for undergoing transfusion (OR 2.79, 95% CI 0.82–9.52, *p* = 0.101).

**Table 4:** Adjusted multivariable analysis (with marginal values and odds ratios) of management, charges, and outcomes in pediatric adenoidectomies by tonsillectomy status

		Adjusted (Tonsillectomy & Adenoidectomy vs. reference Adenoidectomy)	95% Confidence Interval	*p*-value
Total Charges	Charges (Marginal $)	852.06	(-10,211.20 to 11,915.31)	0.880
Length of Stay	Length of Stay (Marginal days)	-0.50	(-1.04 to 0.04)	0.070
Number of Procedures	Procedures (Marginal number)	-0.01	(-0.17 to 0.15)	0.884
Time Until 1st Procedure	Time (Marginal days)	-0.37	(-0.73 to -0.01)	**0.044**
Mortality	Mortality (OR)	n.a.	n.a.	n.a.
Drainage: Ear, Nose, Sinus, Mouth, Throat	Procedure (OR)	0.56	(0.46 to 0.68)	**< 0.001**
Inspection: Ear, Nose, Sinus, Mouth, Throat	Procedure (OR)	0.59	(0.46 to 0.75)	**< 0.001**
Laryngoscopy	Procedure (OR)	0.64	(0.49 to 0.83)	**< 0.001**
Bronchoscopy	Procedure (OR)	0.57	(0.44 to 0.75)	**< 0.001**
Ventilation and Intubation	Procedure (OR)	1.61	(1.03 to 2.50)	**0.037**
Repair: Ear, Nose, Sinus, Mouth, Throat	Procedure (OR)	0.44	(0.28 to 0.72)	**< 0.001**
Control of Bleeding in Head and Neck	Procedure (OR)	8.09	(1.49 to 43.91)	**0.015**
Removal: Ear, Nose, Sinus, Mouth, Throat	Procedure (OR)	0.71	(0.39 to 1.27)	0.249
Imaging	Procedure (OR)	0.56	(0.32 to 0.996)	**0.049**
Transfusion	Procedure (OR)	2.79	(0.82 to 9.52)	0.101
Postprocedural Respiratory Hemorrhage	Complication (OR)	2.71	(0.82 to 8.93)	0.101
Orbital or Intracranial Complications	Complication (OR)	0.06	(0.01 to 0.26)	**< 0.001**

Multivariable analysis with age, sex, race, household income, primary payer status, hospital region, hospital location & teaching status, severity of illness (APRDRG)

*Note* Bolded values indicate statistical significance (*p* < 0.05)

## Discussion

There is a paucity of literature discussing the independent impact of concomitant tonsillectomy on adenoidectomy in pediatric inpatients. In this study, we used KID to provide a national characterization of concomitant tonsillectomy in a large sample of pediatric inpatients undergoing adenoidectomy.

T&A patients had fewer total charges, LOS, and number of procedures undergone than adenoidectomy alone patients on univariate analysis. This discrepancy in healthcare utilization may be explained by the adenoidectomy alone patients being generally sicker and having conditions necessitating longer hospital stays. Specifically, adenoidectomy alone patients were younger (*p* < 0.001) and more likely to have moderate or major loss of function (*p* < 0.001) than T&A patients. Moreover, adenoidectomy alone patients had higher incidences of cystic fibrosis, breathing and swallowing dysfunction, developmental delay, GERD, and congenital malformations including laryngomalacia, tracheomalacia, atrial septal defect, and ventricular septal defect. In addition to these comorbidities, adenoidectomy alone patients also had higher incidence of orbital or intracranial complications than T&A patients. The unequal distribution of these comorbidities and complications was accounted for in a multivariable analysis with severity of illness, a variable that may represent a patient’s state of health more completely than individual comorbidities. This may explain why T&A patients and adenoidectomy alone patients had similar total charges, LOS, and number of procedures undergone on multivariable analyses.

T&A patients had lower odds for undergoing bronchoscopy, laryngoscopy, and imaging on multivariable analyses. Bronchoscopy and laryngoscopy are performed to evaluate the upper airway and identify any abnormalities that may be responsible for breathing and swallowing dysfunction, which T&A patients had lower incidence of [17–19]. Furthermore, imaging studies such as esophagrams are utilized to manage GERD and other abnormalities of the aerodigestive tract, which T&A patients also had lower incidence of [20]. It is possible that T&A patients required fewer diagnostic procedures during their hospital stays for these reasons.

T&A patients had lower odds for undergoing ear, nose, sinus, mouth, or throat inspection and drainage on multivariable analyses. This finding may be explained by T&A patients having lower incidence of otitis media (21.7% vs. 33.1%, *p* < 0.001) than adenoidectomy alone patients. Enlarged adenoids frequently obstruct the Eustachian tubes and cause fluid accumulation in the middle ears [21]. Treatment of any subsequent otitis media commonly involves bilateral myringotomy and ear tube placement [22–24]. Eustachian tube dysfunction has also been suggested to occur at higher frequencies in patients with GERD and congenital cardiopulmonary malformations [24–26]. T&A patients may thus have required inspection and drainage of the ear, nose, sinus, mouth, or throat less frequently than adenoidectomy alone patients.

T&A patients had higher odds for undergoing control of bleeding in the head and neck. Tonsil surgery is associated with intraoperative blood loss and carries a risk of postoperative hemorrhage, especially in older patients [12, 27]. This is consistent with the results of our study, as T&A patients were older than adenoidectomy alone patients.

As with any national database study, we are limited by the accuracy of the database itself. Our inclusion criteria consisted of the appropriate *ICD-10* codes, but KID is an administrative database generated from discharge data and thus may include patient misdiagnoses or fail to report patient diagnoses, which may explain T&A patients having a considerably lower incidence of adenoid hypertrophy (2.7% vs. 58.9%, *p* < 0.001) than adenoidectomy alone patients. KID also reports on inpatient cases instead of on individual patients, so patients with several inpatient stays may be counted more than once. Although comorbidity data are not directly identified in KID, the manual identification of common comorbidities within the cohort via *ICD-10* codes provides valuable comorbidity information. However, we are unable to differentiate between diagnoses made at presentation and diagnoses made after undergoing a procedure, which is another limitation of our study. The implementation of multivariable regression analyses to control for patient demographics, hospital data, and severity of illness allows for improved independent comparison between adenoidectomy alone and T&A patients. We believe that our sample size is large enough to mitigate these potential limitations and that our findings are nationally generalizable to pediatric inpatient adenoidectomy management. Our results will be strengthened and reaffirmed by further studies that have even more specific procedural, complication, and comorbidity data.

## Conclusion

A majority of the pediatric inpatients undergoing adenoidectomy also underwent concomitant tonsillectomy. Overall, adenoidectomy alone patients and T&A patients had similar total charges, length of stay, and number of procedures undergone. T&A patients had lower odds for undergoing ear, nose, sinus, mouth, or throat inspection and drainage, bronchoscopy, laryngoscopy, and imaging than adenoidectomy alone patients. T&A patients also had higher odds for undergoing control of bleeding in the head and neck than adenoidectomy alone patients. Further investigations are warranted to identify the underlying mechanism contributing to these tonsillectomy-dependent disparities in pediatric inpatients undergoing adenoidectomy.

## Declarations

### Presentation Status

Presented as an Oral Presentation at The Combined Otolaryngology Spring Meetings; Dallas, TX, USA; April 27 – May 1, 2022
